# Follicular fluid-derived exosomes rejuvenate ovarian aging through miR-320a-3p-mediated FOXQ1 inhibition

**DOI:** 10.1093/lifemedi/lnae013

**Published:** 2024-03-25

**Authors:** Yu Liu, Hongbei Mu, Yu Chen, Kexin Li, Qiaojuan Mei, Lingjuan Wang, Tianyu Tang, Qiuzi Shen, Huaibiao Li, Ling Zhang, Jing Li, Wenpei Xiang

**Affiliations:** Institute of Reproductive Health, Tongji Medical College, Huazhong University of Science and Technology, Wuhan 430030, China; Department of Gynaecology, Hubei Maternal and Child Health Hospital, Wuhan 430070, China; Institute of Reproductive Health, Tongji Medical College, Huazhong University of Science and Technology, Wuhan 430030, China; Institute of Reproductive Health, Tongji Medical College, Huazhong University of Science and Technology, Wuhan 430030, China; Institute of Reproductive Health, Tongji Medical College, Huazhong University of Science and Technology, Wuhan 430030, China; Institute of Reproductive Health, Tongji Medical College, Huazhong University of Science and Technology, Wuhan 430030, China; Department of Obstetrics and Gynecology, Tongji Hospital, Tongji Medical College, Huazhong University of Science and Technology, Wuhan 430030, China; Institute of Reproductive Health, Tongji Medical College, Huazhong University of Science and Technology, Wuhan 430030, China; Institute of Reproductive Health, Tongji Medical College, Huazhong University of Science and Technology, Wuhan 430030, China; Institute of Reproductive Health, Tongji Medical College, Huazhong University of Science and Technology, Wuhan 430030, China; Institute of Reproductive Health, Tongji Medical College, Huazhong University of Science and Technology, Wuhan 430030, China; State Key Laboratory of Reproductive Medicine, Nanjing Medical University, Nanjing 211166, China; Institute of Reproductive Health, Tongji Medical College, Huazhong University of Science and Technology, Wuhan 430030, China

**Keywords:** exosome, granulosa cells, miRNAs, ovarian aging, follicular fluid

## Abstract

Ovarian aging is mainly characterized by a progressive decline in oocyte quantity and quality, which ultimately leads to female infertility. Various therapies have been established to cope with ovarian aging, among which exosome-based therapy is considered a promising strategy that can benefit ovarian functions via multiple pathways. Here, we isolated and characterized exosomes derived from ovarian follicular fluid and profiled the differential expression patterns of noncoding exosomal RNAs in young and aged women. Treatment with young mouse-derived exosomes efficiently rescued ovarian function in aged mice. The follicular fluid exosomes from young mice and miR-320-3p can also promote the proliferation of ovarian granulosa cells and improve mitochondrial function from old mice *in vitro*. The mechanism may be involve that exosomes transfer miR-320-3p to granulosa cells, and inhibit the expression of FOXQ1. Exosomes also can increase the number of primordial and growing follicles, and improve the developmental ability of oocytes in the old mice *in vivo*. And hnRNPA2B1 controls miR-320-3p entry into exosomes. This work provides insights into the antiaging potential of follicular fluid-derived exosomes and the underlying molecular mechanisms, which may facilitate prevention of ovarian aging and an improvement in female fertility.

## Introduction

The ovary enters the malfunctional aging phase earlier and faster than other organs and systems, and the aging process has attracted extensive attention in the field of reproduction with three-child policy implemented in 2021 in China. Ovarian aging is mainly characterized by a progressive decline in oocyte quantity and quality, which leads to impaired ovarian reserve, a reduced pregnancy rate and live birth rate, and ultimately to the loss of female fertility [1, 2]. According to a new report in *Lancet*, it is estimated that by 2050, 151 countries, including China, will have an extremely declining fertility rate and a clear trend of advanced reproductive age due to improvements in women’s education levels, increased employment pressure, and easier access to contraception [3]. Coupled with the rising infertility rate, the problem of sustaining and enhancing female reproductive health will be crucial in the years to come.

Several clinical therapeutic strategies have been established to improve ovarian function. Primordial follicle *in vitro* activation provides a practical approach for acquiring fully competent oocytes in infertile patients [4], but it is traumatic and its safety needs further verification. Recently, studies regarding stem cell therapies have revealed their potential in improving ovarian functions. Stem cell treatment rescues age-related ovarian damage [5] and partially restores the fertility of premature ovarian failure patients [6]. Stem cells play their role via various mechanisms, among which the paracrine effect of extracellular vesicles, including exosomes, is considered a promising approach that can be applied in multiple fields. Exosomes are bilayer-lipid-membrane extracellular vesicles (EVs) with diameters ranging from 40 to 200 nm [7, 8]. The typical biomarkers of exosomes are transmembrane tetrad proteins, TSG101, and Flotillin-1. Exosomes can be isolated from cell culture fluids and various body fluids, including urine [9], semen [10], saliva [11], ascites [12], blood [13], cerebrospinal fluid [14], and breast fluid [15]. Because they carry a variety of biologically active substances, including DNA, RNA, and so on, exosomes serve as important mediators of intercellular communication, delivering intracellular contents from their cell of origin to target cells [16]. Previous studies have shown that injection with exosomes isolated from young mouse serum can reverse the expression pattern of aging-associated molecules in the lungs and liver [17]. Importantly, intravenously injected extracellular vesicles from neonatal umbilical cord rejuvenated aged bone marrow–derived mesenchymal stem cells [18]. Similarly, age-related cognitive decline can be restored after the systemic administration of young blood plasma into aged mice [19]. However, whether exosomes from follicular fluid can rescue diminished ovarian function remains unknown.

Bovine follicular fluid-derived exosomes contribute to improvements in the developmental capacity of oocytes [20]. Equine follicular fluid-derived exosomes serve as key regulators in follicle maturation [21]. Exosome-secreted miRNAs can regulate the maturation of oocytes and sperm [22]. Wei et al. [23] found that miRNAs are crucial for the implementation of exosome functions, and the lipid membrane structure of exosomes can act as a protective barrier to prevent RNase-induced miRNA degradation [24]. Animal studies have shown that exosomal miRNA expression profiles in follicular fluid are significantly correlated with age. Compared with follicular fluid exosomes from young mares, exosomes from aged mares carried higher levels of miRNAs, eventually leading to impaired maturation of oocytes [21, 25]. Sohel et al. [26] found certain differences in exosomal miRNA expression patterns in follicular fluid containing mature and immature oocytes. Furthermore, high-throughput sequencing demonstrated differentially expressed miRNA profiles between young and aged women [27]. All this evidence demonstrates that the follicular fluid exosomal miRNA expression profile is closely correlated with age and ovarian function, indicating the possible involvement of exosomes in ovarian aging; thus, the specific underlying mechanisms need to be further explored.

In this study, we established an miRNA expression profile in human follicular fluid-derived exosomes, revealed the therapeutic potential of follicular fluid exosomes to counteract ovarian aging, identified the key regulator miR-320-3p, and unveiled the corresponding mechanism underlying miR-320-3p-mediated regulation of ovarian functions, thus providing powerful evidence for establishing a novel and promising strategy to improve ovarian function in aged women.

## Results

### Characteristics of follicular fluid-derived exosomes

To investigate the characteristics of follicular fluid-derived exosomes, we isolated follicular fluid-derived exosomes (FF-exos) from young (≤ 30-year-old, Y-exos) and aged (> 38-year-old, O-exos) women (Fig. S1A) and identified them by transmission electron microscopy (TEM), nanoparticle tracking analysis (NTA) and western blot analyses. The TEM results showed the circular membrane structure of isolated vesicles (Fig. S1B). The number and size distribution of Y-exos and O-exos were determined by NTA, showing that the exosome diameters mainly ranged from 50 to 150 nm (Fig. S1C). Moreover, no significant differences in terms of particle size and concentration were observed between Y-exos and O-exos (Fig. S1D and S1E). We further performed western blot analysis to examine exosome-specific markers to validate the enrichment of exosomes (Fig. S1F). Collectively, these data demonstrated the main characteristics of Y-exos and O-exos and showed no significant difference between these two groups.

### Y-exos can enhance functions of aged mouse-derived GCs *in vitro*

To investigate whether Y-exos could be internalized by aged mouse-derived granulosa cells (GCs), we first isolated exosomes and GCs from young and aged mice, respectively. The purity of isolated GCs was determined by its specific marker FSHR (Fig. S2A), while that of the extracted exosomes was evaluated by TEM, NTA and western blot analysis. On the basis of TEM imaging, these exosomes exhibited typical circular membrane vesicles (Fig. S2B). The particle size distribution and concentration of the exosomes were assessed via NTA, and the exosomal size ranges from 50 to 150 nm (Fig. S2C and S2D). Western blot analysis showed that the exosomal surface markers TSG101 and FLOT1 could be detected in the exosome samples (Fig. S2E). Next, the uptake of Y-exos by ovarian GCs of aged mice was observed *in vitro*. As shown in Fig. S2F, PKH67-labeled Y-exos could be observed in the GCs at 24 h after co-culture, suggesting that Y-exos can be internalized by GCs.

**Figure 1.**
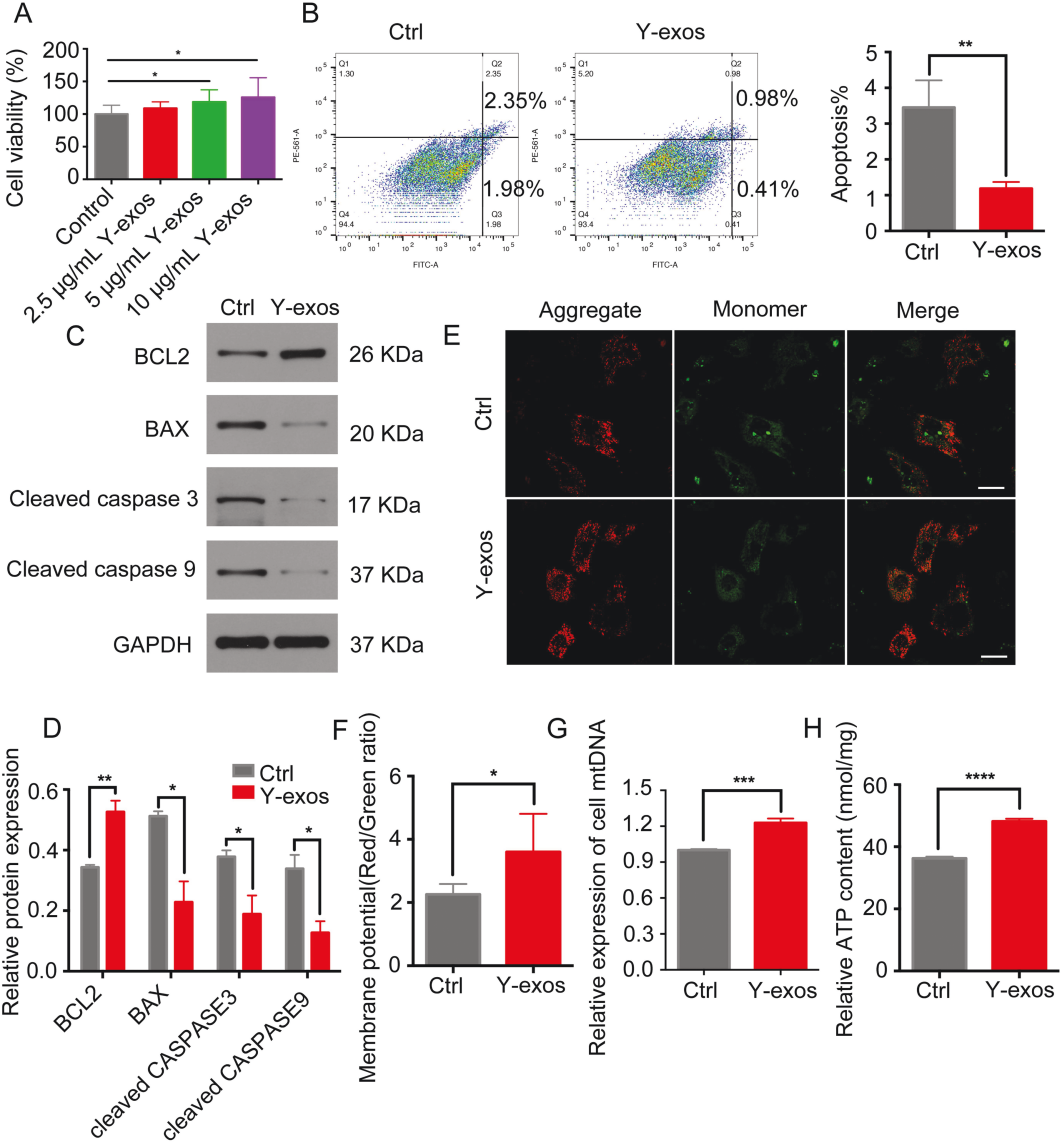
**Y-exos promote proliferative capacity of aged mice derived GCs *in vitro*.** (A) Cell viability analyzed by CCK8 assay. (B) Apoptotic rate of aged mice derived GCs with or without Y-exos treatment was determined by flow cytometry. (C, D) Cleaved caspase-3, cleaved caspase-9, BAX, and BCL2 protein expression were analyzed by western blot. (E, F) MMP assessed by JC-1 staining. Scale bar, 100 μm. (G) The relative expression of cell mtDNA. (H) ATP concentration in GCs was analyzed.

To examine the effects of Y-exos on GC functions, ovarian GCs of aged mice were exposed to Y-exos at different concentrations (2.5, 5, 10 μg/mL) for 24 h. Y-exos markedly enhanced cell viability, and this effect appeared to be concentration dependent (*P *< 0.05, Fig. 1A). Ultimately, we chose 5 μg/mL Y-exos for subsequent experiments. Annexin V staining indicated a significant decrease in apoptosis in the Y-exos group (*P *< 0.01, Fig. 1B). We also observed a downregulation in cleaved caspase3, cleaved caspase9, and BAX protein levels and a substantial upregulation in BCL2 protein levels in the Y-exos group (*P *< 0.05, Fig. 1C and 1D). In terms of mitochondrial function in GCs, we found higher mitochondrial membrane potential (MMP), ATP levels and mitochondrial DNA (mtDNA) copy numbers after Y-exos treatment (Fig. 1E–H). Collectively, these results indicate that Y-exos can promote proliferation and improve mitochondrial functions in aged mouse-derived GCs.

### Y-exos can rescue age-related ovarian dysfunction in vivo

**Figure 2.**
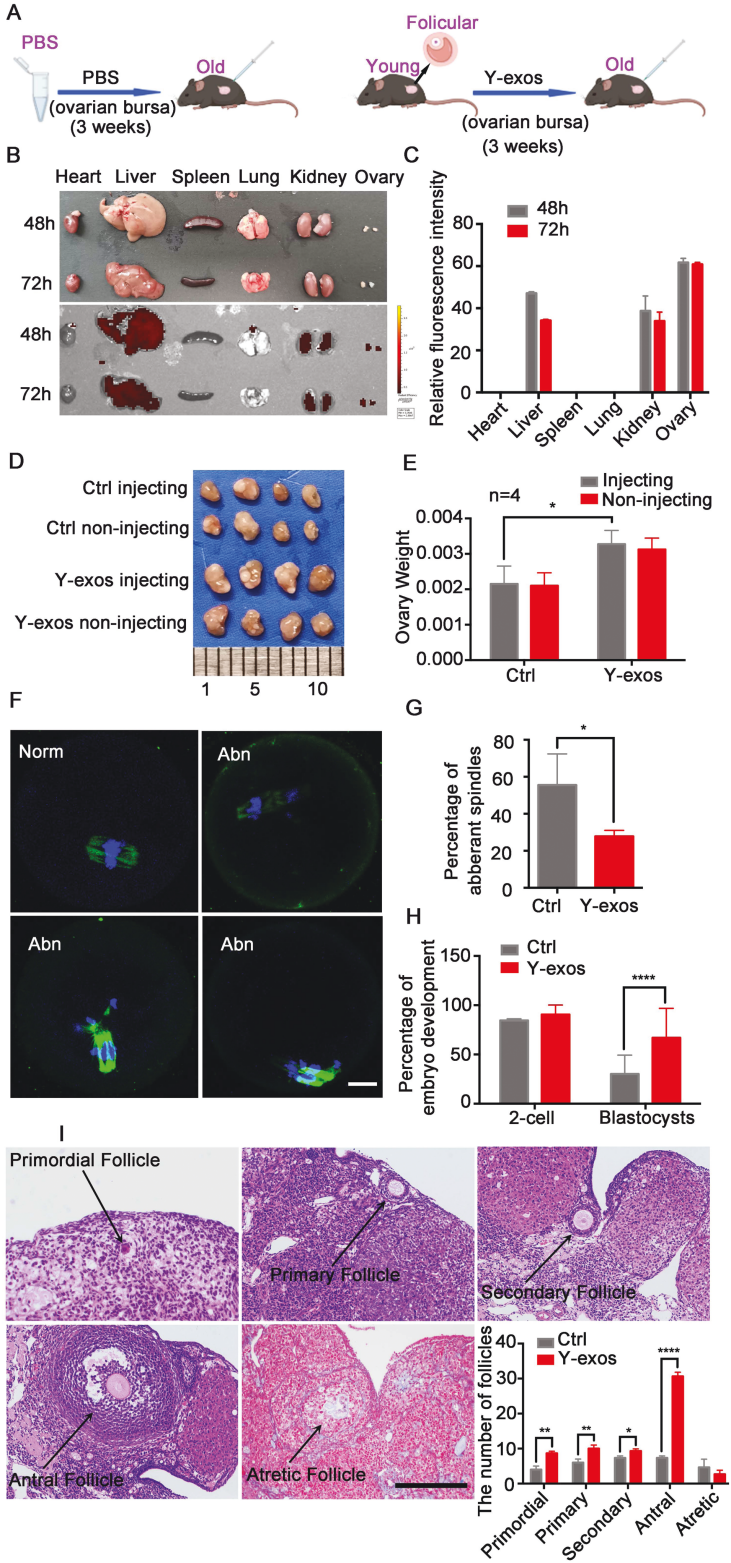
**Y-exos can rescue age-related ovarian dysfunction *in vivo*.** (A) Schematic illustration of ovarian bursa injection of follicular fluid exosomes from young mice (Y-exos, right) and PBS (left) into old mice for 3 weeks. (B) Representative fluorescence distribution imaging of PKH67-labeled Y-exos in different organs of mice after 48 h and 72 h of injection. (C) Quantitation of fluorescence intensities in different organs after 48 h and 72 h of injection. (D) Ovaries at 3 weeks after injection. Isolated ovaries from control (Ctrl) versus Y-exos treatment. (E) Comparison of ovarian weight between control and Y-exos treatment (*n* = 4). (F) Representative image of MII oocytes with normal (Norm) or aberrant (Abn) spindles in control and Y-exos treatment groups. Scale bar, 100 μm. (G) Percentage of aberrant spindles in control and Y-exos treatment groups. *, *P* < 0.05. (H) Percentage of embryo development between two groups. Ctrl, *n* = 41, Y-exos, *n* = 42. ****, *P* < 0.0001. (I) Representative ovarian histology images and distribution of different stages of follicles with or without Y-exos treatment. Scale bar, 100 μm. *, *P* < 0.05; **, *P* < 0.01; ****, *P* < 0.0001.

To determine whether Y-exos treatment can rescue age-related ovarian dysfunction *in vivo*, forty-week-old mice received unilateral ovarian bursa local injection with Y-exos (Fig. 2A). Fluorescence signals were observed in the liver, kidney and ovary, with the highest fluorescence intensity appearing in the ovary on both sides (Fig. 2B and 2C), suggesting that exosomes can be transferred to mouse ovaries, the liver and the kidney after ovarian bursa local injection. Intriguingly, we found that unilateral injection did not cause a difference in the volume of the ovaries on both sides within the same mouse (Fig. 2B).

We next observed the effects of Y-exos on mouse ovaries *in vivo*. The ovarian volume and weight after Y-exos treatment were increased compared to those in the control group (*P *< 0.05, Fig. 2D and 2E), but no difference was observed between the injected side and the noninjected side (Fig. 2D and 2E). TUNEL assay results indicated a decreased apoptotic rate in the Y-exos group (Fig. S3A and S3B). We also analyzed the expression levels of aging-related signaling factors, such as *Mmp9*, *P53*, *Il-1α*, *P21*, *Il-8*, *Mmp1*, *Ccl20* and *P16* via real-time qPCR. As shown in Fig. S3C, these factors were efficiently downregulated in the Y-exos-treated ovaries, while the expression levels of telomerase-related genes, including *Terf2*, *Tep1*, *Men1*, *Mre11a*, *Tert*, and *Tnks*, increased significantly (Fig. S3D). We next performed TEM analysis to demonstrate the ultrastructural change in ovaries after exosome treatment. No significant changes in either mitochondrial density or abnormal mitochondrial number were observed (Fig. S3E and S3F). However, the number of lipid drops in GCs was found to be significantly decreased in the Y-exos-treated group (Fig. S3E and S3G). Taken together, our results suggest that Y-exos treatment can partially rescue age-related ovarian dysfunction.

To further evaluate the effects of Y-exos on ovarian function, we collected GCs and examined the changes in mitochondrial function. After Y-exos treatment, both MMP and intracellular ATP production were efficiently improved (Fig. S3H and S3I), while mtDNA copy number exhibited a slight elevation (Fig. S3J), demonstrating that Y-exos treatment can enhance mitochondrial functions in aged mice.

Next, MII oocytes were collected for oocyte quality analysis. Immunofluorescence staining of β-tubulin in MII oocytes showed a lower rate of aberrant spindles in the Y-exos-treated group than in the control group (Fig. 2F and 2G), suggesting that Y-exos treatment can alleviate age-related meiosis disorder to a certain extent. We then performed *in vitro* fertilization (IVF) to determine the impact of Y-exos on oocyte developmental potential. Although the 2-cell rate did not exhibit an obvious change after Y-exos treatment (89.92 ± 0.06 *vs.* 92.00 ± 0.10), the blastocyst rate in the Y-exos group was significantly improved compared to that in the control group (66.98 ± 0.30 *vs.* 30.21 ± 0.19) (Fig. 2H), indicating that the developmental competence of MII oocytes was improved after Y-exos administration. In addition, in the Y-exos group, we found that follicles at different developmental stages were obviously increased, while atretic follicles were reduced (Fig. 2I). Surprisingly, the antral follicle count in the Y-exos-treated group had a nearly 3-fold increase compared to that in the control group, suggesting that Y-exos treatment largely improved follicular development (Fig. 2I). There were no difference in the number of female (*P* ＞ 0.05, Fig.S3K) in the Y-exos group, however, the pups per female have an increasing trend (*P* ＞ 0.05, Fig. S3L).

### miRNA sequencing revealed differentially expressed miRNAs in follicular fluid-derived exosomes between young and aged women

**Figure 3.**
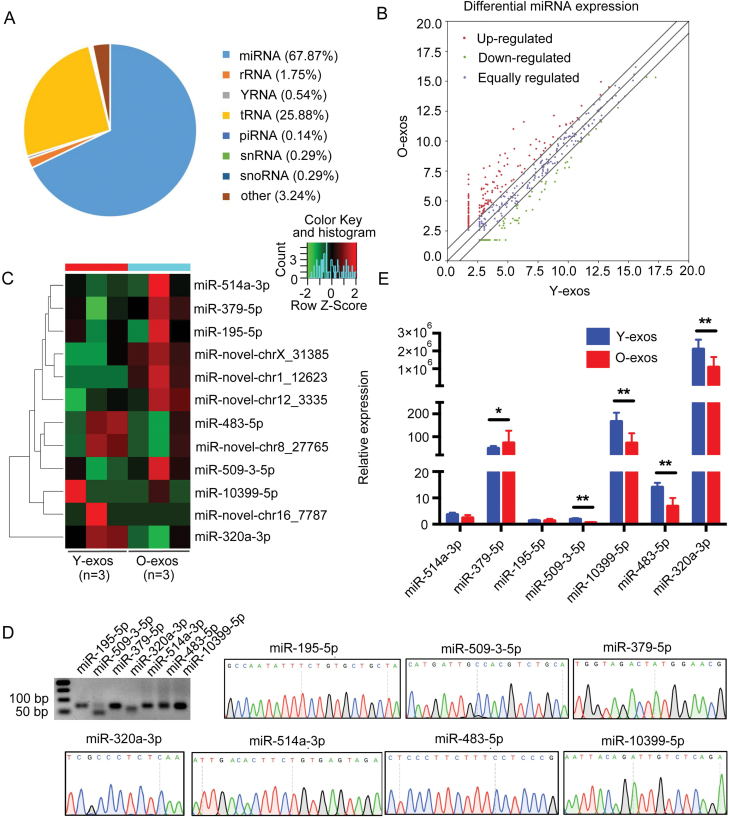
**Noncoding RNA sequencing revealed differentially expressed miRNAs in follicular fluid-derived exosomes between young and old women.** (A) Pie chart representation of distribution of small RNA categories in Y-exos and O-exos. (B) Volcano plot showing the different expression patterns of miRNAs in Y-exos and O-exos. (C) Heatmap of differentially expressed miRNAs in Y-exos and O-exos. (D) The specificity of miRNA primers was examined by 2% agarose gel electrophoresis and Sanger sequencing. (E) The expression of seven differentially expressed miRNAs in a larger cohort (*n* = 36) were examined by RT-qPCR. *, *P* < 0.05; **, *P* < 0.01.

To further investigate the mechanism underlying the beneficial effects of Y-exos in the ovaries of aged, we analyzed the miRNA transcriptome of human Y-exos and O-exos via high-throughput sequencing analysis. In Y-exos and O-exos, 67.87% of reads were aligned to miRNAs (Fig. 3A), and a total of 637 miRNAs were identified (Fig. 3B). Among all the identified miRNAs, 12 miRNAs exhibited significantly different expression levels between Y-exos and O-exos, with 7 miRNAs upregulated and 5 miRNAs downregulated (*P *< 0.05, Fig. 3C). Agarose gel electrophoresis and Sanger sequencing were first performed to verify the specificity of the miRNA primers (Fig. 3D), and then, these verified primers were used for subsequent RT‒qPCR analysis to validate the sequencing data. As shown in Fig. 3E, miR-320a-3p was markedly upregulated in the Y-exos group and had a significantly higher level than other miRNAs, including miR-10399-5p and miR-483-5p. Therefore, we speculate that miR-320a-3p may play a key role in exosome-mediated effects on ovarian functions.

### miR-320-3p enhances mouse GCs proliferation and mitochondrial functions in vitro

**Figure 4.**
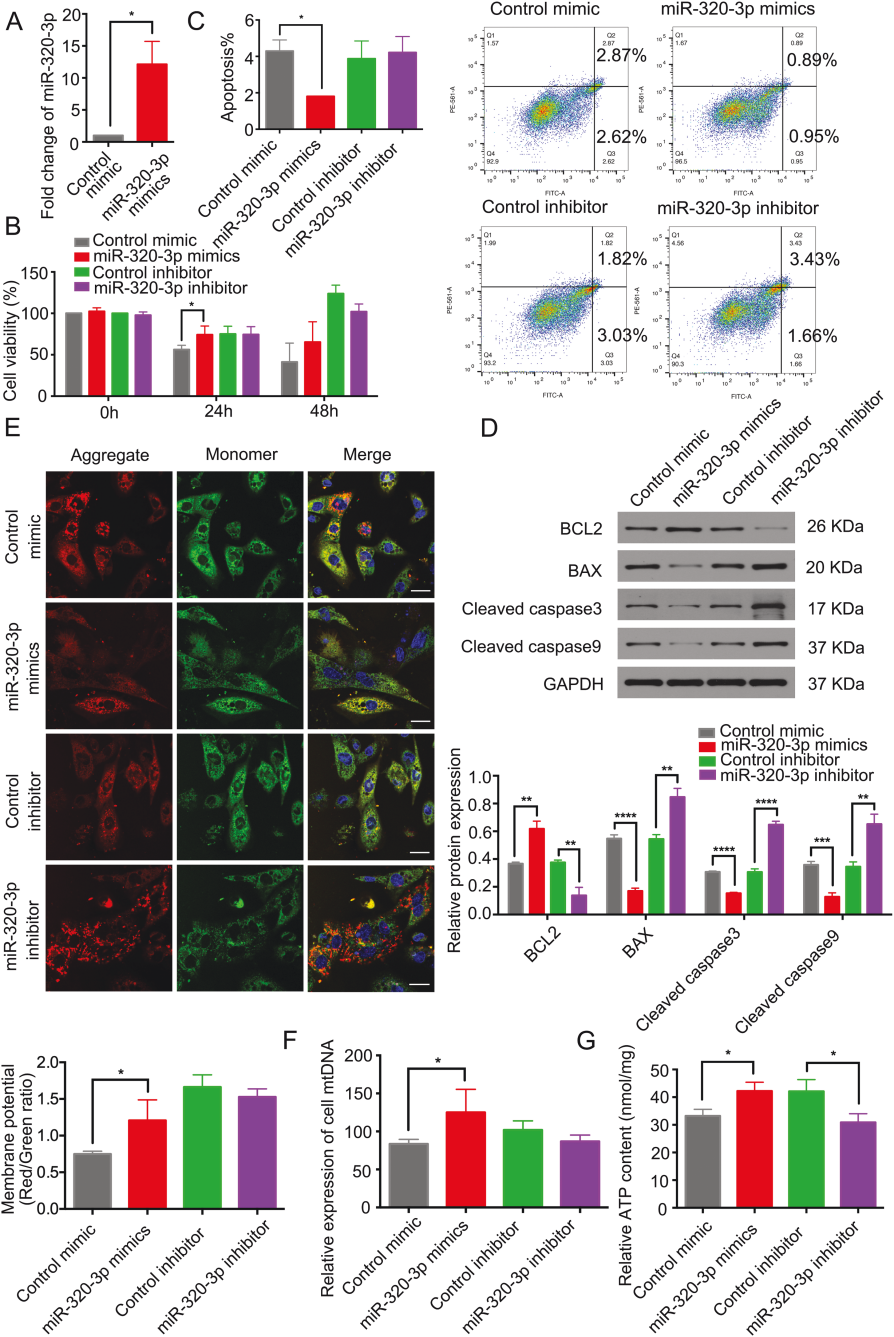
**miR-320-3p enhances mouse GCs proliferation and mitochondrial functions *in vitro.***(A) RT-qPCR of miR-320-3p in cells transfected with control and miR-320-3p mimics. *, *P *< 0.05. (B) Cell viability analyzed by CCK8 assay in miR-320-3p mimics, miR-320-3p inhibitor or control. (C) Apoptotic rate of aged mice derived GCs was determined by flow cytometry. (D) Cleaved caspase-3, cleaved caspase-9, BAX, and BCL2 protein expression were analyzed by western blot. (E) MMP assessed by JC-1 staining. Scale bar, 100 μm. (F) The relative expression of cell mtDNA. (G) ATP concentration in GCs was determined.

To examine whether exosomal miR-320-3p is responsible for the exosome-mediated effects, we transfected GCs with miR-320-3p mimic and confirmed the transfection efficiency via RT‒qPCR (Fig. 4A). miR-320-3p mimic enhanced cell viability (*P *< 0.05, Fig. 4B), and decreased cell apoptosis (*P *< 0.05, Fig. 4C) and downregulation cleaved caspase3, cleaved caspase9, BAX expression and upregulation BCL2 protein levels in GCs (*P* < 0.05, Fig. 4D), whereas the miR-320-3p inhibitor had the opposite effect on GCs (Fig. 4B, 4C and 4D). These results suggest that miR-320-3p can promote proliferation and enhance resistance against apoptosis in GCs. We next detected the changes in mitochondrial functions upon miR-320-3p overexpression or inhibition and found that miR-320-3p overexpression improved MMP, enhanced mtDNA expression and promoted ATP production (Fig. 4E–G). These results suggest that miR-320-3p can enhance GCs proliferation and mitochondrial functions *in vitro*.

### Follicular fluid-derived exosomes transfer miR-320-3p to GCs derived from aged mice

**Figure 5.**
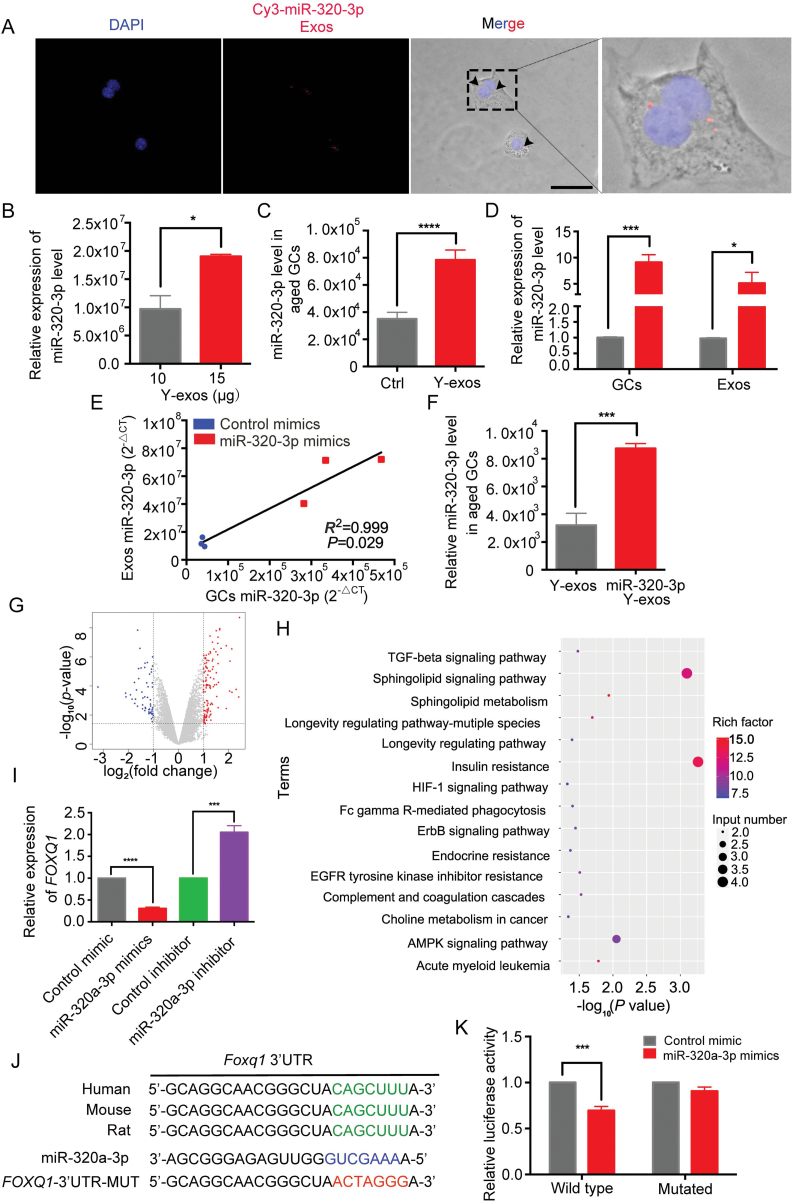
**Follicular fluid-derived exosomes transfer miR-320-3p to aged mice derived GCs and miR-320a-3p directly binds to *Foxq*1 to regulate GCs proliferation.** (A) Existence of Cy3-labeled miR-320-3p in aged mouse-derived GCs, which were co-culture with Y-exos isolated from young mice derived GCs transfected with Cy3-labeled miR-320-3p mimics. Scale bar, 100 μm. (B) miR-320-3p level measured with different amount of young follicular fluid exosomes. *, *P* < 0.05. (C) Relative level of miR-320-3p in aged mice derived GCs co-culture with Y-exos. ****, *P* < 0.0001. (D, E) Coordinated changes of miR-320-3p level in the cultured young mice derived GCs and their secreted exosomes. Gray bars represent the control group, red bars represent the miR-320-3p mimics group. Line represents linear regression. *, *P* < 0.05; ***, *P* < 0.001. (F) Relative level of miR-320-3p in aged mice derived GCs co-culture with Y-exos isolated from young mice derived GCs transfected with miR-320-3p mimics. (G) Differentially expressed genes upon miR-320a-3p overexpression profiled by RNA-seq were illustrated by the volcano plot. (H) KEGG pathway enrichment analysis of possible miR-320a-3p target genes. (I) *Foxq1* mRNA quantitation by RT-qPCR in COV434 cells line after transfection with miR-320a-3p mimics, inhibitor or control. ***, *P* < 0.001; ****, *P* < 0.0001. (J) Schematic representation of putative miR-320a-3p binding sites on 3ʹUTR of *Foxq1* in human, mouse and rat. (K) COV434 cells line transfected with wild-type or mutated *Foxq1* 3'UTR luciferase constructs and control mimic or miR-320a-3p mimics. ***, *P *< 0.001.

Since the pro-proliferation effect of Y-exos and miR-320-3p had been verified *in vitro*, we next tried to determine whether miR-320-3p mediates the Y-exos-induced effects in GCs. We first isolated exosomes containing Cy3-labeled miR-320-3p from GCs of young mice and then cocultured them with GCs from aged mice. After co-culturing for 24 h, the Cy3-labeled miR-320-3p fluorescence signal was observed in GCs (Fig. 5A), suggesting that exosomal miR-320-3p was absorbed by GCs. Then, we found that the level of miR-320-3p increased as the number of exosomes increased (*P *< 0.0001, Fig. 5B). Co-culture with Y-exos increased the miR-320-3p level in GCs from aged mice (*P *< 0.05, Fig. 5C), further validating that miR-320-3p can be transferred to GCs via exosomes. By overexpressing miR-320-3p in cultured GCs, we found that the miR-320-3p levels in exosomes were increased (*P *< 0.05, Fig. 5D), and the miR-320-3p levels in exosomes exhibited a positive correlation with those in GCs (*P *< 0.05, Fig. 5E). Moreover, the level of miR-320-3p in GCs was further increased after co-culture with exosomes from GCs overexpressing miR-320-3p (Fig. 5F).

### miR-320a-3p directly binds to Foxq1 to regulate GCs proliferation

To determine which genes are under the control of miR-320-3p, we performed RNA-seq analysis to identify the differentially expressed genes upon miR-320a-3p overexpression, and 56 genes were identified as downregulated genes (defined as fold change ≥1, *P* < 0.05, Fig. 5G) in the miR-320a-3p mimic group. We then performed KEGG enrichment analysis to further identify the possible targets of miR-320a-3p. Among all the enriched KEGG terms, “longevity regulating pathway-multiple species” and “longevity regulating pathway” were strongly correlated with the phenotype that we characterized in previous results (Fig. 5H). Combined with the results of the functional screening of differentially expressed genes, we eventually identified the following five genes as possible targets of miR-320a-3p that may contribute to the miR-320a-3p-induced effects: forkhead box Q1 (*Foxq1*), ribosomal protein S6 kinase B2 (*RPS6KB2*), protein phosphatase 2 regulatory subunit B’beta (*PPP2R5B*), nuclear receptor subfamily 1 group H member 2 (*NR1H2*), and insulin-like growth factor binding protein 5 (*IGFBP5*). Then, we analyzed the mRNA expression levels of the above five genes in the miR-320a-3p mimic and miR-320a-3p inhibitor groups and found that these candidate genes were downregulated in the miR-320a-3p mimic group and upregulated in the miR-320a-3p inhibitor group (Figs. 5I and S4A). However, only *Foxq1*, *PPP2R5B*, and *IGFBP5* were recognized as biological targets of miR-320a-3p by TargetScan. Subsequently, a dual luciferase reporter assay was performed to validate the interaction between miR-320a-3p and its target transcripts (Fig. 5J and 5K, S4B, S4C and S4D, S4E). Considering the sequence conservation across species and the functions of these candidate genes, we selected *Foxq1* as the target of miR-320a-3p for further validation studies.

### miR-320a-3p overexpression promotes GCs proliferation by mediating FOXQ1 downregulation

To examine whether FOXQ1 regulates proliferation, FOXQ1 was knocked down in aged mouse-derived GCs (Fig. S5A and S5B), resulting in a significant increase in the viability of old GCs (*P *< 0.05, Fig. S5C). We then examined the apoptosis level and the protein expression levels of cleaved caspase3, cleaved caspase9, BAX, and BCL2. The rate of apoptosis was decreased (*P *< 0.05, Fig. S5D), with cleaved caspase3, cleaved caspase9 and BAX downregulated, and BCL2 upregulated in the *Foxq1*-siRNA group (*P *< 0.05, Fig. S5E). FOXQ1 knockdown increased MMP levels, enhanced ATP production and elevated cell mtDNA levels (*P *< 0.05, Fig. S5F–S5H). These results suggest that FOXQ1 mediates the pro-proliferative and anti-apoptotic effects of miR-320-3p in GCs.

**Figure 6.**
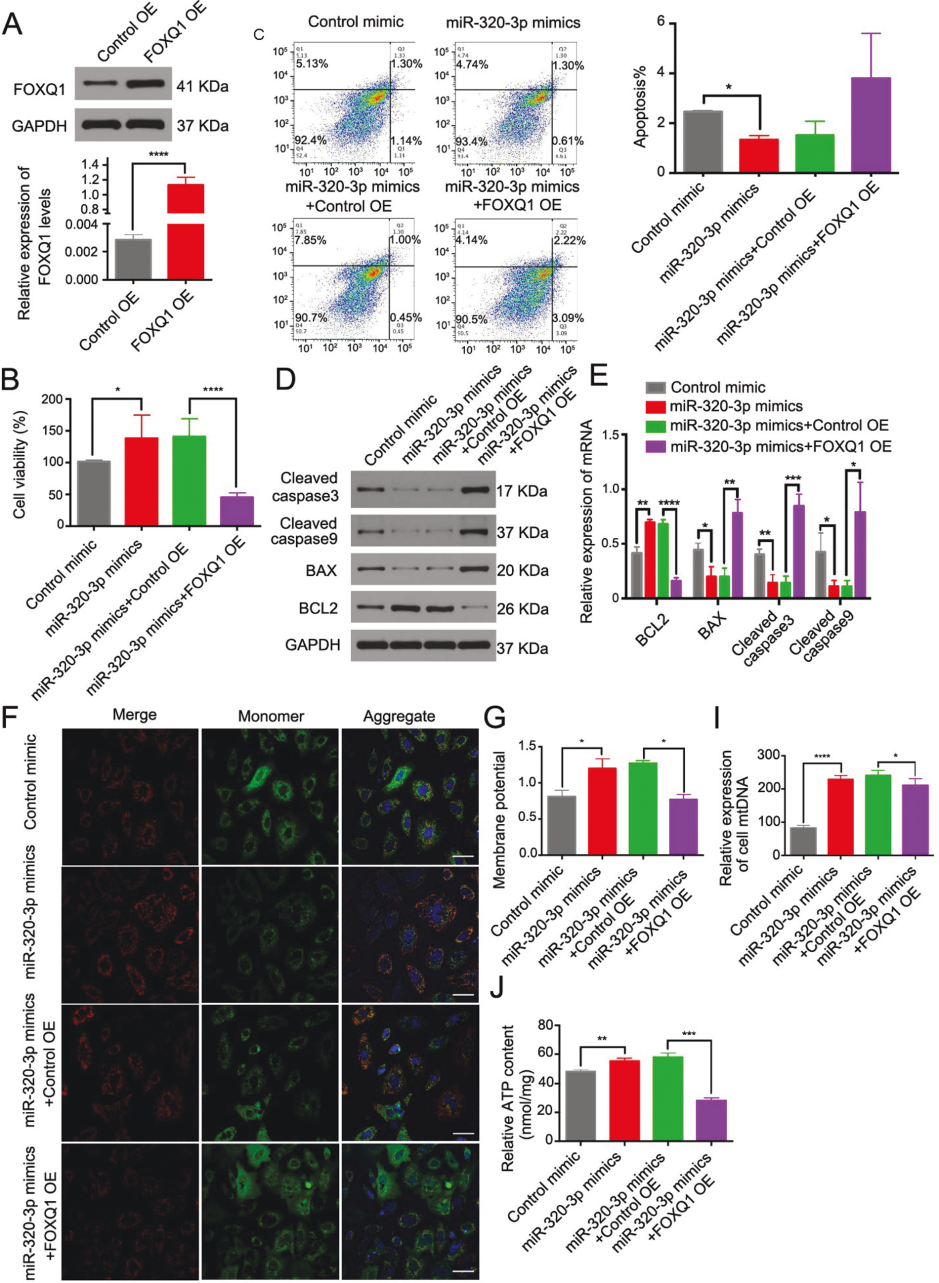
**miR-320a-3p overexpression promotes GCs proliferation by mediating FOXQ1 downregulation.**(A) Western blot and RT-qPCR results of FOXQ1 levels in FOXQ1-overexpressed cells or its control. ****, *P *< 0.0001. (B) Cell viability analyzed by CCK8 assay in cells of the control, miR-320-3p mimics, miR-320-3p mimics + control OE and miR-320-3p mimics + FOXQ1 OE groups. OE: overexpression. (C) Apoptotic rate of aged mice derived GCs was determined by flow cytometry. *, *P *< 0.05. (D) Cleaved caspase-3, cleaved caspase-9, BAX, and BCL2 protein expression were analyzed by Western blot. (E) and summary graph. (F) MMP assessed by JC-1 staining. Scale bar, 100 μm. (G) and quantitative graph. *, *P *< 0.05. (H) ATP concentration in aged mice derived GCs. **, *P *< 0.01; ***, *P *< 0.001. (I) The relative expression of cell mtDNA. ****, *P *< 0.0001; *, *P *< 0.05.

We next analyzed whether miR-320-3p regulates GCs functions by targeting *Foxq1*. The overexpression efficiency was first determined by western blot and RT‒qPCR analyses (Fig. 6A). Then, we analyzed whether FOXQ1 overexpression could at least partially impede the miR-320-3p-induced effects in GCs. We found that FOXQ1 overexpression disturbed the pro-proliferative and anti-apoptotic effects (Fig. 6B–E) and hindered the enhancement of mitochondrial functions mediated by miR-320-3p overexpression (Fig. 6F–I). These results demonstrate that the effects of miR-320-3p in GCs are achieved by targeting the *Foxq1* transcript.

### hnRNPA2B1 controls the loading of miR-320-3p into exosomes

**Figure 7.**
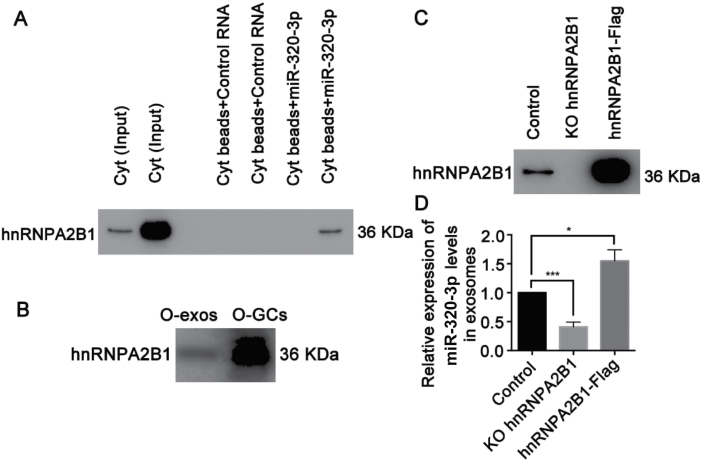
**hnRNPA2B1 controls the loading of miR-320-3p into exosomes.** (A) RNA pull-down western blot analysis showing the binding of hnRNPA2B1 to miR-320-3p in Y-Cyt and O-Cyt. Y-Cyt: young mice cytoplasm, O-Cyt: old mice cytoplasm. (B) Representative Western blot analysis showing the expression of hnRNPA2B1 in O-exos and O-GCs. O-exos: old mice derived exosomes, O-GCs: old mice derived GCs. (C) Western blot analysis of hnRNPA2B1 in HeLa cells from KO or overexpression hnRNPA2B1. KO: knockout, hnRNPA2B1-Flag: overexpression hnRNPA2B1. (D) RT-qPCR analysis of miR-320-3p in exosomes from KO or overexpression hnRNPA2B1. *, *P *< 0.05; ***, *P *< 0.001.

To investigate the molecular mechanism by which miR-320-3p is loaded into exosomes, an RNA pull-down assay was performed, and proteins bound with miR-320-3p were subjected to mass spectrometry for identification (Table S1). The precipitated proteins included various heterogeneous nuclear ribonucleoproteins (hnRNPs), including hnRNPA2B1, hnRNPL, hnRNPR, and hnRNPU. We then confirmed the specific binding between hnRNPA2B1 and miR-320-3p (Fig. 7A) and examined the level of hnRNPA2B1 protein expression in exosomes and in GCs from aged mice (Fig. 7B).

To reveal the role of hnRNPA2B1 in the process of miR-320-3p loading into exosomes, we knocked out and overexpressed hnRNPA2B1 in HeLa cells (Fig. 7C) and measured miR-320-3p levels in both exosomes and GCs. We found that hnRNPA2B1 depletion led to a significant reduction in miR-320-3p in exosomes (*P *< 0.05, Fig. 7D). In contrast, hnRNPA2B1 overexpression resulted in an elevation in exosomal miR-320-3p levels (*P *< 0.05, Fig. 7C and 7D), suggesting that hnRNPA2B1 is indispensable for miR-320-3p loading from GCs to exosomes.

## Discussion

The intercellular communication between GCs and oocytes provides nutrients for the development and maturation of oocytes [28], and oocyte-secreted factors promote the proliferation and differentiation of GCs [29]. GCs dysfunction impairs oocyte developmental capacity and quality. In the co-culture experiment of exosomes and the mouse oocyte-cumulus complex, the type of cells absorbing exosomes was clarified. Only cumulus GCs in the oocyte-cumulus complex absorbed exosomes, while oocytes did not. This may be attributed to the zona pellucida surrounding the oocytes blocking the transmission of exosomes.

We evaluated how the behaviors of GCs isolated and cultured *in vitro* from aged mice were affected by follicular fluid exosomes from young mice. The results showed that follicular fluid exosomes from young mice could be absorbed by ovarian GCs of aged mice, significantly enhancing the proliferation ability and mitochondrial function of GCs and alleviating GCs apoptosis. Next, *in vivo* experiments revealed that young mouse-derived exosomes significantly increased the weight of mouse ovaries and the number of follicles at all developmental stages in aged mice, attenuated apoptosis in ovarian tissue, enhanced the mitochondrial function of GCs, alleviated aging-related demonstrations and significantly reduced the number of lipid droplets in ovarian follicles of aged mice. The accumulation of intracellular lipids leads to high levels of free fatty acids that cause oxidative damage and produce highly active oxidative metabolites that cause irreversible cell damage [30], and long-term damage and oxidative processes impair cellular homeostasis, ultimately leading to apoptosis [31]. The function of GCs is closely related to oocyte quality, and enhancement of GCs function improves the quality of oocytes. This may be part of the mechanism by which young mouse-derived exosomes improve the ovarian function of aged mice.

Furthermore, transplantation of follicular fluid exosomes from young mice reduced the number of abnormal spindles and increased the percentage of blastocysts in aged mice. The results of mating experiments showed that young follicular fluid exosomes have an increasing litter size trend, validating the conclusion that young mouse-derived exosomes may improve oocyte quality and ovarian function in aged mice. Consistent with our results, intravenous injection of serum exosomes from young mice reversed the increase in the expression of aging-related markers in aged male mice [17]. Another study found that intraovarian injection of exosomes from human umbilical cord mesenchymal stem cells increased the number and quality of oocytes and restored diminished fertility in aged female mice [32]. These findings confirm that exosomes can modulate ovarian functions after being absorbed by the ovaries.

Current evidence has shown that exosomes contain a large number of miRNAs. These miRNAs can be transferred to target cells via exosomes, thus changing the gene expression and biological activity of target cells [33–35]. In this study, higher levels of miR-320a-3p were found in follicular fluid exosomes from young women, indicating that loading disorder of miRNAs into exosomes may occur during the ovarian aging process. We found that miR-320-3p can be transferred by young mouse-derived exosomes to the ovarian GCs of aged mice. miR-320-3p was found to be involved in regulating GCs proliferation, apoptosis, and mitochondrial functions. Furthermore, miR-320-3p overexpression and co-culture with young mouse-derived exosomes exerted similar effects on ovarian GCs of aged mice, suggesting that follicular fluid-derived exosomes regulate GCs function by delivering miR-320-3p.

miRNAs are short noncoding RNA molecules that act as gene regulators by inhibiting translation or inducing the degradation of target mRNAs via binding to the 3’UTR of target mRNAs [36–39]. miR-320a-3p is a member of the miR-320a family. In acute pancreatitis, miR-320-3p promotes cell proliferation by inhibiting the inflammatory response through the PTK2 axis [40]. Furthermore, miR-320-3p facilitates myogenic progenitor proliferation and differentiation by regulating actin remodeling [41]. In addition, a recent study found that miR-320-3p regulates hypoxia-induced proliferation, migration and apoptosis of pulmonary artery smooth muscle cells [42]. These findings suggest that upregulation of miR-320-3p may promote cell proliferation.

Several target genes of miR-320a-3p have already been identified, among which *Foxq1*, a human-mouse homologous gene, may be responsible for the effect of miR-320a-3p in regulating the function of ovarian GCs in aged mice. The interaction between miR-320a-3p and *Foxq1* was validated by dual luciferase experiments, indicating that the positive effect of exosomal miR-320-3p may, at least partially, rely on suppressing FOXQ1 expression. These results link follicular fluid exosomal miR-320-3p to *Foxq1*, providing new clues for unveiling the impact of young follicular fluid exosomes on aged ovarian GCs.

Selective loading of miRNAs into exosomes is directly related to pathological processes in several diseases, including cancer, neuroinflammation, diabetes, and heart disease [43]. Various mechanisms have been indicated in the loading of miRNAs into extracellular vesicles, including cytosol-mediated miRNA delivery, specific modifications and the interaction between miRNAs and RNA-binding proteins [44]. Previous studies have confirmed that RNA-binding proteins play an important role in the loading of miRNAs into exosomes. In hepatocytes, the RNA-binding protein SYNCRIP controls the sorting and loading of miRNAs into hepatocyte exosomes [45]. In lung cancer cells, miR-122-5p is selectively secreted into lung cancer exosomes by binding to the RNA-binding protein hnRNPA2B1 [46]. In the present study, hnRNPA2B1 was identified as a miR-320-3p binding protein. hnRNPA2B1 is responsible for controlling the sorting and packaging of miRNAs into exosomes by binding to specific motifs [47]. Here, we revealed the interaction between miR-320a-3p and the RNA-binding protein hnRNPA2B1 and elucidated that hnRNPA2B1 depletion can lead to a disorder in miR-320-3p loading into exosomes, partially revealing the molecular mechanisms underlying the process of miRNA loading into follicular fluid-derived exosomes. However, whether and how hnRNPA2B1 is involved in regulating the biological functions of follicular fluid-derived exosomes needs further exploration.

Compared with other RNA therapies, exosomes have been considered an ideal vehicle for drug delivery. As a natural product of the human body, follicular fluid exosomes have the advantages of low immunogenicity, low ethical risk, nontumorigenicity and high safety. Moreover, the double-membrane structure of exosomes prevents their contents from being degraded. The biggest challenge in RNA therapy is its cytotoxicity. In this study, co-culture with follicular fluid exosomes and miR-320-3p overexpression in GCs exerted similar effects on ovarian function in aged mice, suggesting that exosomes are a promising strategy for RNA therapy.

**Figure 8.**
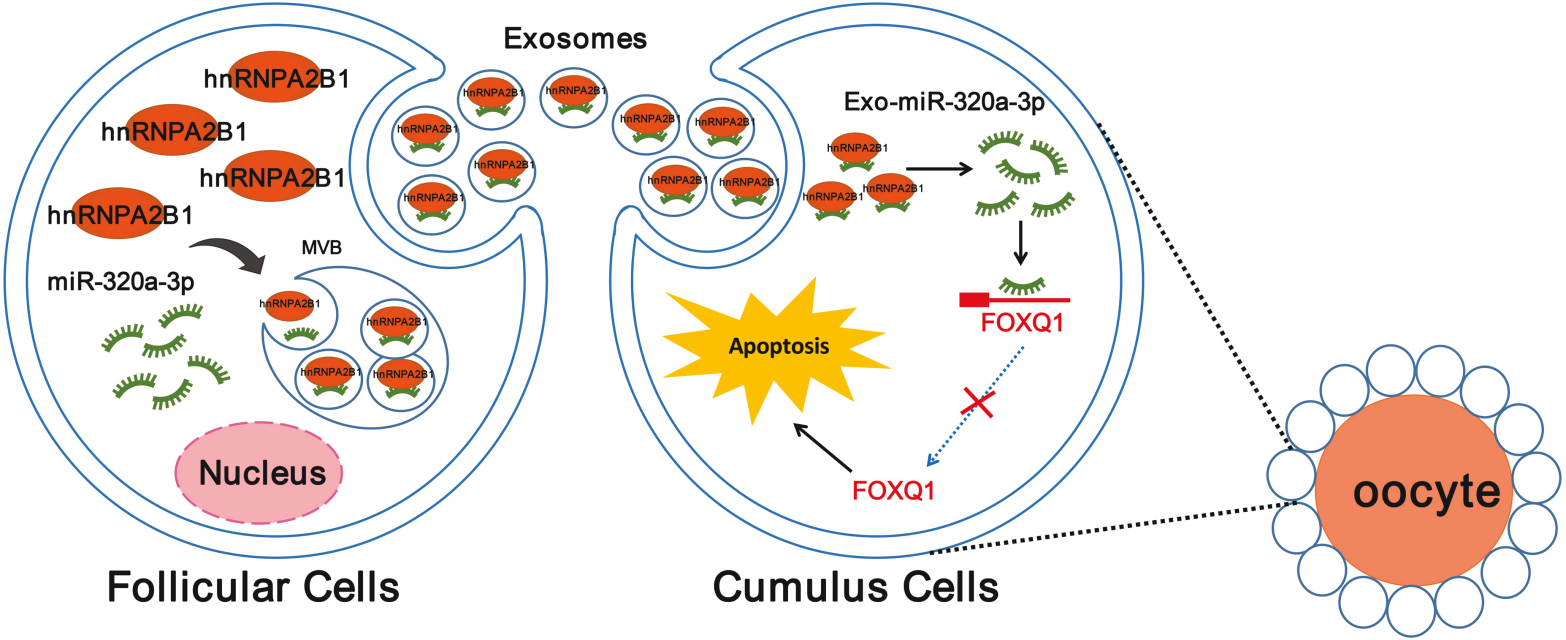
**Schematic diagram.** hnRNPA2B1 mediated miR-320a-3p sorting into young exosomes, which inhibited aged granulosa cells apoptosis, promoted cells proliferation, and improved mitochondrial function by inhibiting FOXQ1 expression of aged mouse *in vitro.*

In conclusion, these results confirmed that follicular fluid-derived exosomes of young mice can promote ovarian functions by transferring miR-320a-3p to GCs (Fig. 8), indicating that follicular fluid-derived exosomes can be considered a potential therapy for ovarian aging. Follicular fluid is an unneeded clinical product generated during the process of assisted reproductive technology. Compared with exosomes derived from other cells, follicular fluid exosomes have the advantages of easy accessibility and high yield, which gives them promising clinical application prospects. Our research results verify the antiaging effects of follicular fluid exosomes from young patients and provide a highly valuable treatment strategy to counteract ovarian aging.

## Research limitations

There are still some limitations to this study. First, the present study did not further evaluate the exact pathway mechanism by which miR-320-3p regulates FOXQ1 expression to promote ovarian GC proliferation and enhance mitochondrial function in older aged mice. In addition, the contents of follicular fluid exosomes are complex, and it cannot be excluded that some other factors also play important biological functions, such as lncRNAs, circRNAs, or proteins. That may be helpful in the future screening of key regulatory molecules.

## Methods

### Extraction of follicular fluid-derived exosomes

Human follicular fluid samples were collected from follicles that reached a diameter of 18 mm or more by ultrasound inspection. The samples were centrifuged at 300 *g* for 10 min to remove dead cells, cell debris, and large EVs and then at 2000 *g* for 10 min and 10,000 *g* for 30 min. Then, the samples were filtered through a 0.22 μm filter, and the supernatants were ultracentrifuged and washed with phosphate-buffered saline (PBS) at 100,000 *g* for 90 min. The exosome precipitate was suspended in 100 μL of PBS.

Mouse follicular fluid-derived exosomes were extracted by mechanical method. Specifically, 6- to 8-week-old C57BL/6J female mice were treated with 10 IU pregnant mare serum gonadotropin (PMSG) at 46−48 h prior to exosome extraction. After execution, mouse ovaries were then dissected and placed in Dulbecco's Modified Eagle Medium (DMEM)/F12 complete medium to remove blood and fat tissue. Ovaries were then transferred to a fresh DMEM/F12 complete medium and the ovarian follicles were punctured with 1 mL syringes to release the follicular fluid. The follicular fluid was then filtered using a 70 μm filter to remove oocytes and remaining ovarian tissues. The centrifugation steps were performed at 4°C, including 300 *g* for 10 min, 2000 *g* for 10 min, and 10,000 *g* for 30 min, and the supernatant was collected after each centrifugation step. The filtrate was then passed through a 0.22 μm filter and subjected to EV extraction using the ExoQuick-TC™ reagent kit according to the manufacturer’s instructions. The EVs were resuspended in 100 μL of PBS, frozen at −80°C, and identified using electron microscopy, Western blot, and NTA, as described in the protocol for human follicular fluid EV identification.

### Exosome uptake assay

Exosomes were stained with the green fluorescent cell membrane labeling dye PKH67 (Sigma, USA) according to the manufacturer’s instructions at room temperature and then blocked with 1% bovine serum albumin (BSA)/PBS solution. An ExoQuick-TC™ kit (SBI, USA) was used to extract exosomes and remove the unconjugated dye. Labeled exosomes were incubated with GCs for 24 h. After treatment, GCs were collected, and nuclei were stained with Hoechst 33342 (Invitrogen, USA). The fluorescence signal was observed using a fluorescence microscope.

### Intrabursal injection of exosomes

C57BL/6J mice (40 weeks) were used for modeling, and 1% pentobarbital was used for anesthesia. The skin near the ovary side adjacent to the spine was shaved, a small incision was made, and the fat pad at the ovary was fixed to expose the ovary. Intracapsular ovary injection was performed, and the modeling time was 3 weeks. The mice were sorted into a PBS injection group and a Y-exos (young C57BL/6J mouse follicular fluid exosomes) injection group. The injection volume was 10 µL, and the exosome mass was 20 µg. In the ovarian weight experiment, unilateral injection of ovaries (injected side and noninjected side) was performed, and in the other experiments, bilateral injection of ovaries was performed.

### Research ethics

This project was approved by the Ethics Committee of Reproductive Medicine Center, Tongji Medical College, Huazhong University of Science and Technology ([2020] ethical approval number: 007) and Institutional Animal Care and Use Committee, Huazhong University of Science and Technology (ethical approval number: S3745). Follicular fluid samples were collected with the informed consent of patients.

### Statistical analysis

Data are expressed as the mean ± standard deviation (SD). The results between two groups that conformed to a normal distribution were compared using unpaired *t* tests and those that did not conform to a normal distribution were compared via Mann‒Whitney tests. Results among three or four groups were compared via one-way analysis of variance. Statistical analysis was performed using the Statistical Package for the Social Sciences, version 12.0 (SPSS Inc., Chicago, IL, USA). *P *< 0.05 indicates that a difference is statistically significant. Each experiment was repeated three times.

## Supplementary Material

lnae013_suppl_Supplementary_Figures_S1

lnae013_suppl_Supplementary_Figures_S2

lnae013_suppl_Supplementary_Figures_S3

lnae013_suppl_Supplementary_Figures_S4

lnae013_suppl_Supplementary_Figures_S5

lnae013_suppl_Supplementary_Data

## Data Availability

The data that support the findings of this study are available from the corresponding author upon reasonable request.
