# Supplementary figures and images for: Follicular fluid-derived exosomes rejuvenate ovarian aging through miR-320a-3p-mediated FOXQ1 inhibition
Source: Life Med. 2024 Mar 25;3(1):lnae013. doi: 10.1093/lifemedi/lnae013 (PMC11749233; doi:10.1093/lifemedi/lnae013)

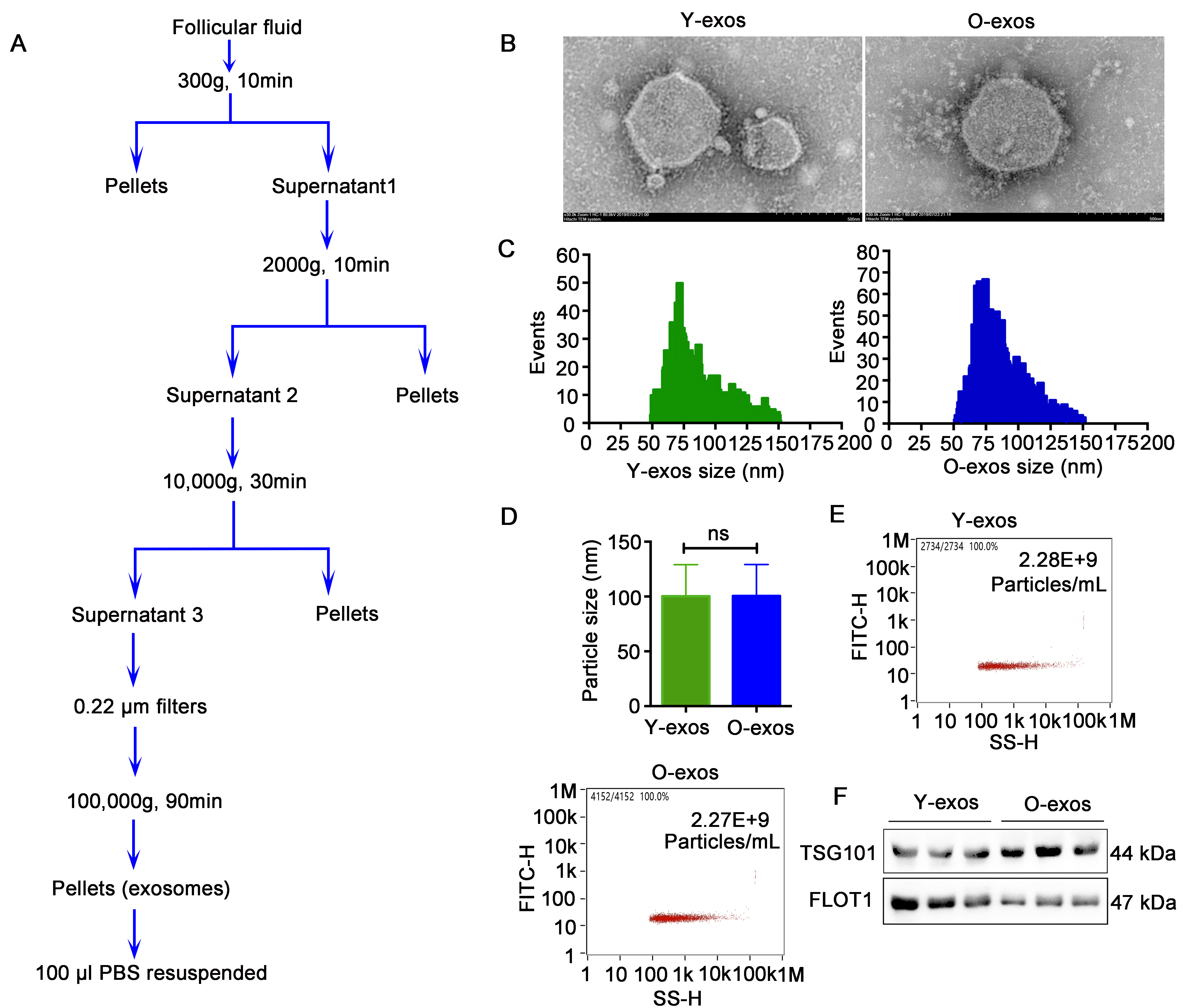

Supplement: lnae013_suppl_Supplementary_Figures_S1 [file lnae013_suppl_Supplementary_Figures_S1.tif]

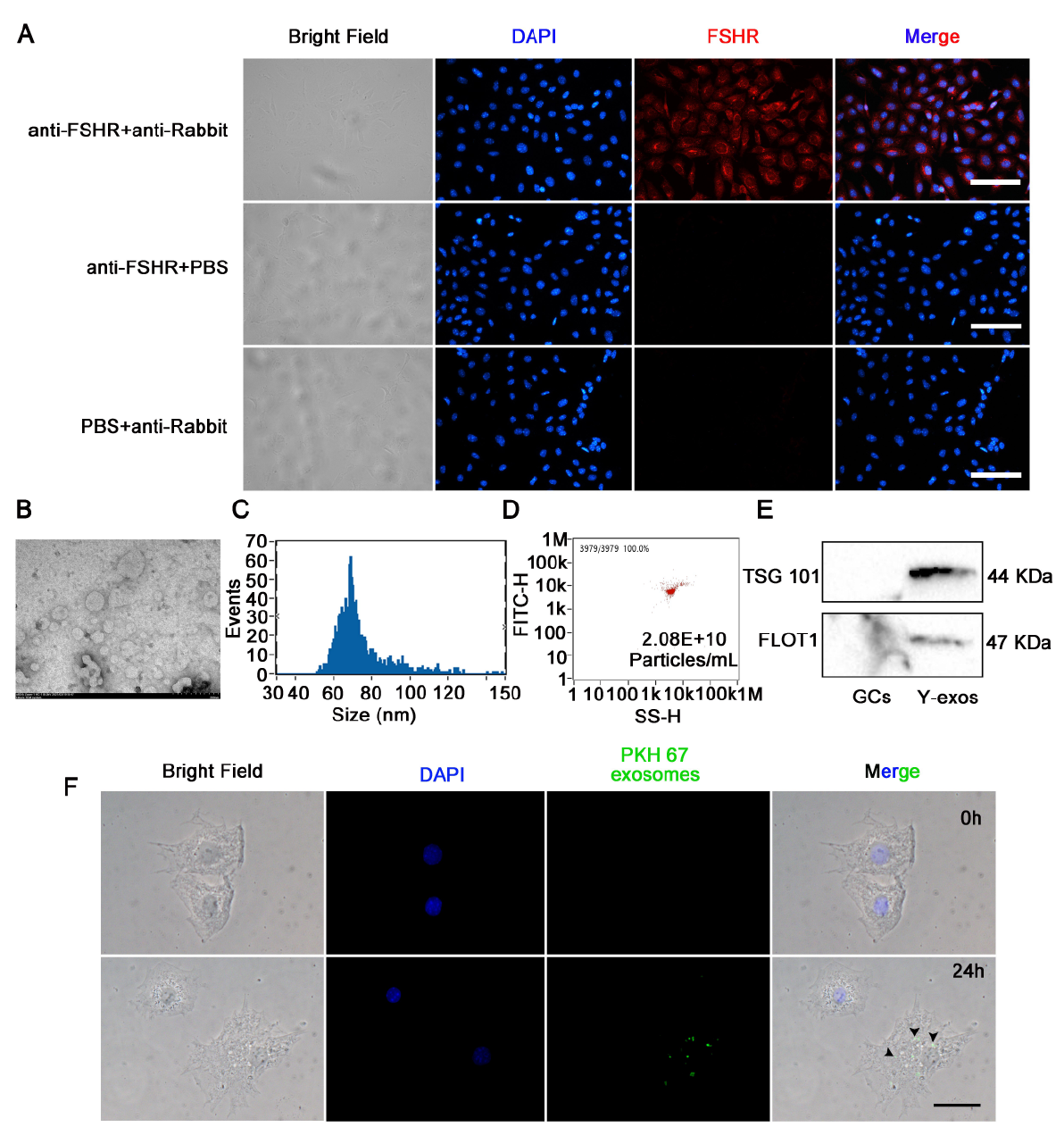

Supplement: lnae013_suppl_Supplementary_Figures_S2 [file lnae013_suppl_Supplementary_Figures_S2.tif]

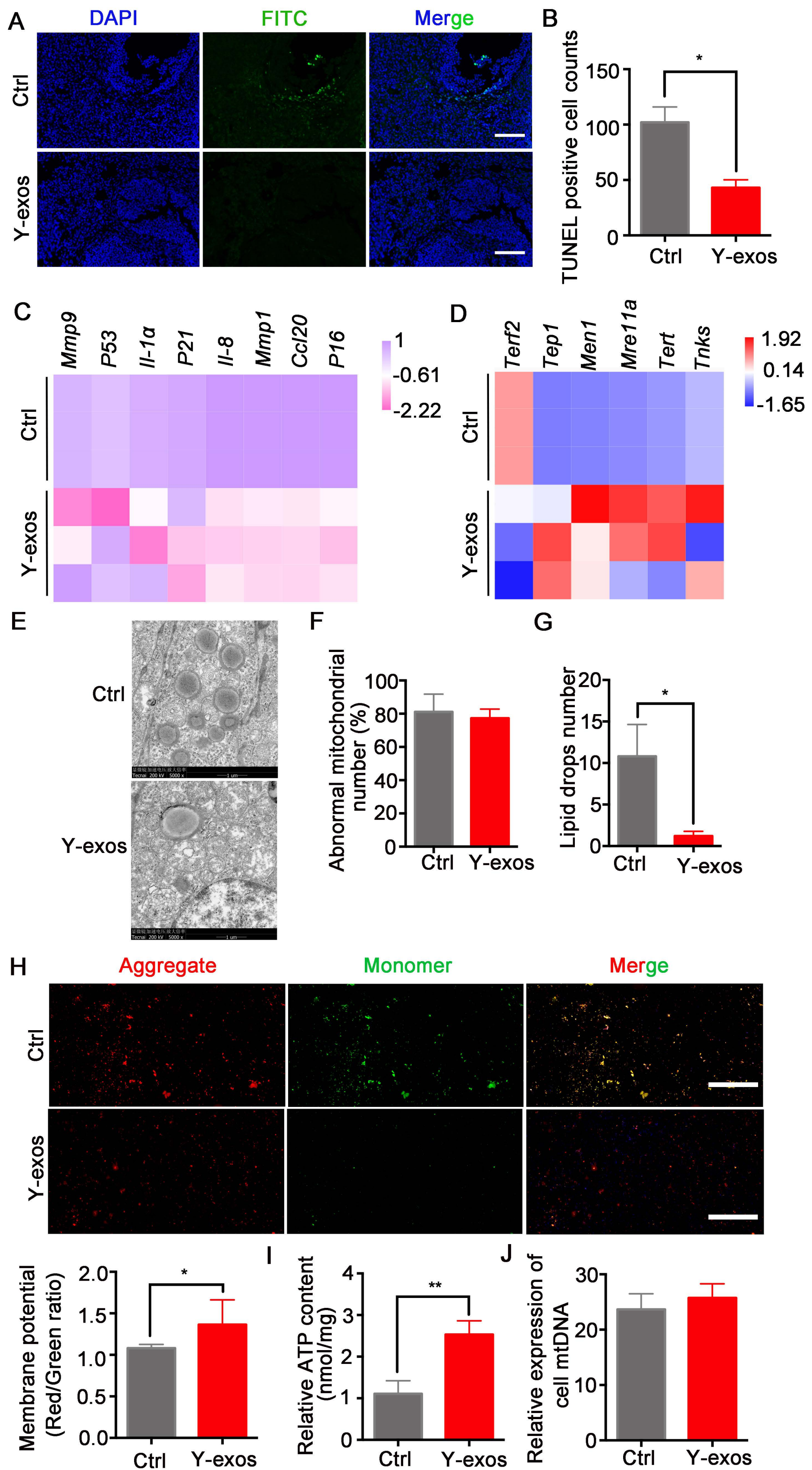

Supplement: lnae013_suppl_Supplementary_Figures_S3 [file lnae013_suppl_Supplementary_Figures_S3.pdf]

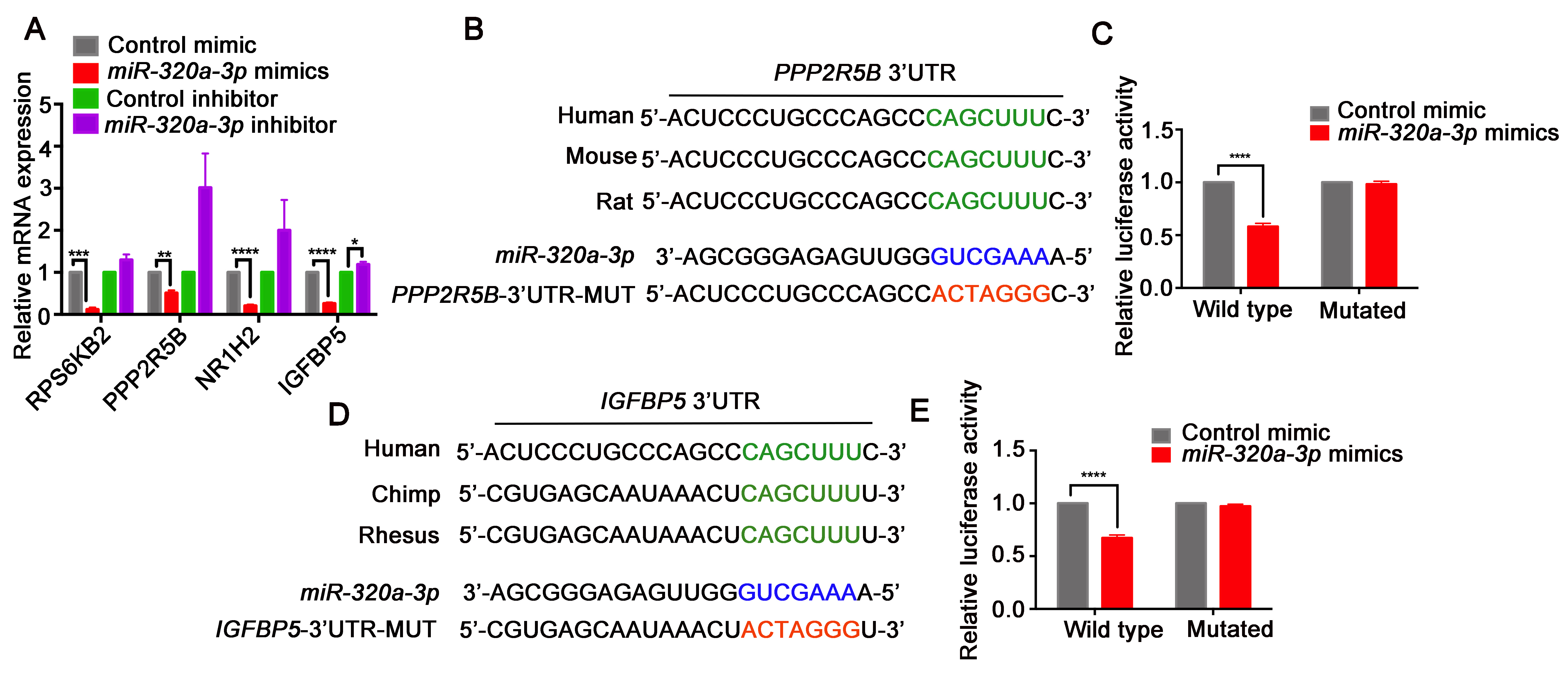

Supplement: lnae013_suppl_Supplementary_Figures_S4 [file lnae013_suppl_Supplementary_Figures_S4.tif]

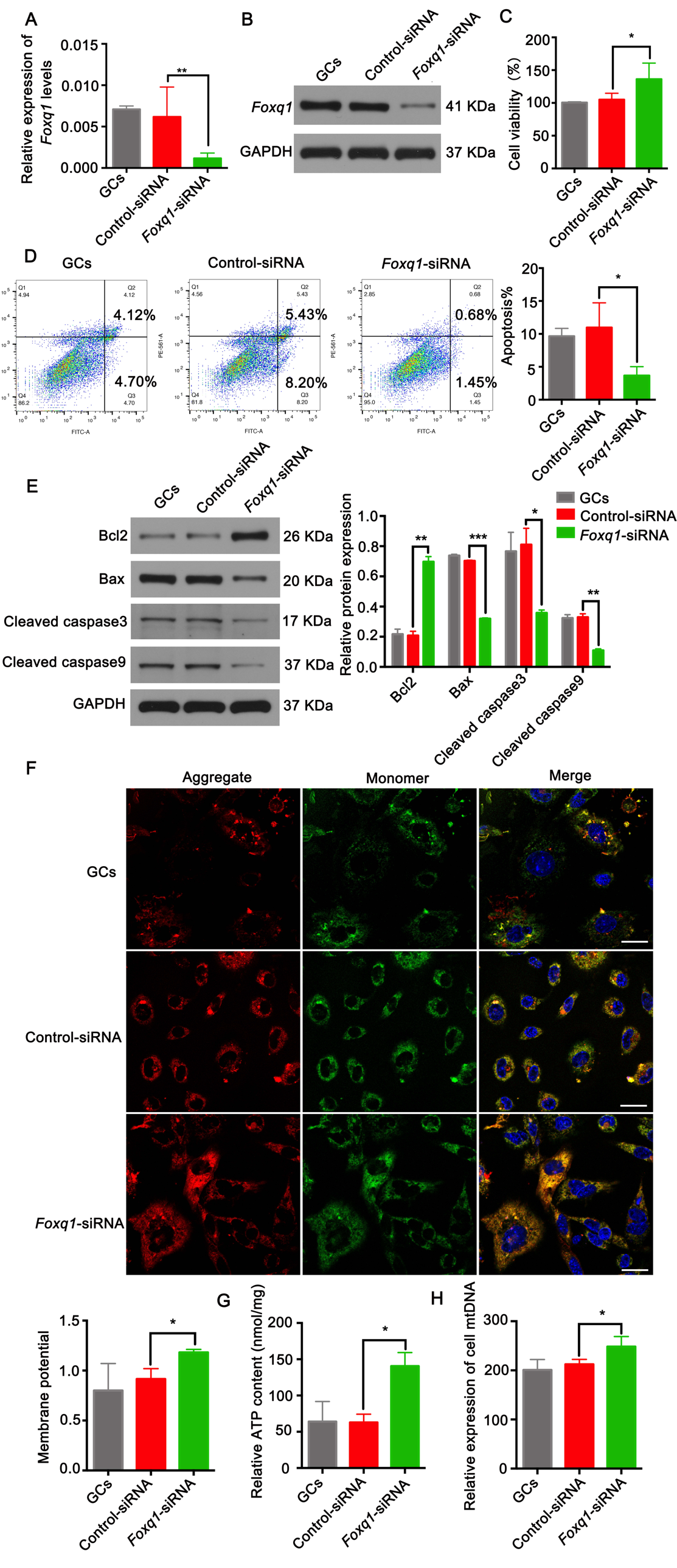

Supplement: lnae013_suppl_Supplementary_Figures_S5 [file lnae013_suppl_Supplementary_Figures_S5.pdf]
