# Supplementary material for: Follicular fluid-derived exosomes rejuvenate ovarian aging through miR-320a-3p-mediated FOXQ1 inhibition
Source: Life Med. 2024 Mar 25;3(1):lnae013. doi: 10.1093/lifemedi/lnae013 (PMC11749233; doi:10.1093/lifemedi/lnae013)
Supplement: lnae013_suppl_Supplementary_Data [file lnae013_suppl_Supplementary_Data.docx]

Follicular fluid-derived exosomes rejuvenate ovarian aging through miR-320a-3p-mediated FOXQ1 inhibition.

Yu Liu^1,2,#^, Hongbei Mu^1,#^, Yu Chen^1^, Kexin Li^1^, Qiaojuan Mei^1^, Lingjuan Wang^3^, Tianyu Tang ^1^, Qiuzi Shen^1^, Huaibiao Li^1^, Ling Zhang^1,^*, Jing Li^4,^*, Wenpei Xiang^1,^*

^1^Institute of Reproductive Health, Tongji Medical College, Huazhong University of Science and Technology, Wuhan 430030, China

^2^Department of Gynaecology, Hubei Maternal and Child Health Hospital, Wuhan 430070, China

^3^Department of Obstetrics and Gynecology, Tongji Hospital, Tongji Medical College, Huazhong University of Science and Technology, Wuhan 430030, China

^4^State Key Laboratory of Reproductive Medicine, Nanjing Medical University, Nanjing 211166, China

^#^These authors contributed equally to this work.

^*^Correspondence: Zhling312@163.com (L.Z.), ljwth@njmu.edu.cn (J.L.), wpxiang2010@hust.edu.cn (W.X.)

**Materials and methods**

**Sample collection**

For this study, 36 women undergoing IVF or ICSI at the Center for Reproductive Medicine of Tongji Medical College at the Huazhong University of Science and Technology were recruited from December 2019 to January 2021. The young exosome group (Y-exos, *n* = 23) was defined as ≤ 30 years old, and ovarian function was normal, while the old exosome group (O-exos, *n* = 13) was defined as ≥ 38 years old. The ovarian stimulation regimen for all patients involved was the antagonist protocol. Midstream follicular fluid samples were collected, without visible blood or culture medium. The exclusion criteria were women with cancer, endometriosis, or other reproductive endocrine diseases and infectious diseases.

**Isolation of mouse granulosa cells (GCs)**

According to previous research ^1^, primary mouse ovarian GCs were collected from 40-week-old C57BL/6J female mice. Briefly, the mice were injected intraperitoneally with 10 IU mare serum gonadotropin (PMSG) (Sansheng Pharmaceutical Co., Ltd., Ningbo, China). After 46–48 h, the mice were killed by cervical dislocation. The ovarian tissue and oocytes were removed through a 70 μm filter. Ovarian GCs were collected and placed in DMEM/F12 (HyClone, USA) complete medium supplemented with 10% FBS (*v*/*v*, Gibco, USA), 100 IU/ml penicillin and 100 μg/ml streptomycin (Gibco) at 37°C with 5% CO_2_.

**Identification of GCs**

Mouse GCs (5 × 10^5^) were seeded in 6-well plates, cultured under standard conditions for 48 h, and then fixed in 4% (*v*/*v*) paraformaldehyde for 20 min at room temperature in the dark. Triton X-100 (0.2%) was added to 6-well plates for 10 min. Then, the GCs were incubated with follicular stimulating hormone receptor (FSHR) primary antibody at 4°C overnight and then with a secondary anti-rabbit IgG (Aspen, USA) at room temperature for 1 h. Finally, the GCs were stained with DAPI for 5 min. PBS was used as a negative control for primary and secondary antibodies.

**Transmission electron microscopy (TEM)**

Mouse GCs or exosomes were suspended and fixed in 2.5% glutaraldehyde at 4°C overnight. GCs and exosomes were fixed on a carbon-coated copper grid and then stained with 2% uranyl acetate solution for 2 minutes. The samples were observed with a transmission electron microscope.

**Quantification of exosomes by nanoparticle tracking analysis (NTA)**

Nanoparticle tracking analysis (NTA) was performed using a ZetaView® Nanoparticle Tracking Analyzer PMX-120 instrument. The data were analyzed using ZetaView Analyze software.

**Concentration of exosomes**

The concentration of exosomes was detected with a BCA Protein Assay Kit (Beyotime, China) according to the manufacturer's protocol. Exosomes (5 μL) were first lysed with 1.25 μL 5 × RIPA buffer and 5 × protease inhibitor in tubes. Then, 5 μL exosomes and 200 μL BCA solution were added to 96-well plates. The plate was incubated for 30 min at 37°C, and the absorbed light was detected at 562 nm with a microplate reader.

**RNA sequencing analysis**

Exosomes were isolated from young and aged human follicular fluid and processed for miRNA-sequencing analysis using an Illumina HiSeq4000 platform. Total RNA was extracted using TRIzol reagent (Invitrogen, USA). The RNA concentration was detected with a NanoDrop ND-1000 spectrophotometer (Thermo Fisher Scientific, Waltham, USA). Total RNA from each sample was used to prepare the miRNA sequencing library. The adaptor sequences were trimmed, and the adaptor-trimmed reads (≥ 15 nt) were collected using cutadapt software (v1.9.2). A multiple change > 2.0 and a *P* value < 0.05 were used as the thresholds for differential miRNA screening.

**Quantitative real-time PCR**

Total RNA was isolated from cells with TRIzol (Invitrogen, USA). HiScript II qRT Super Mix (Vazyme, China) was used to synthesize the first strand of cDNA. Quantitative real-time PCR was performed using Taq Pro Universal SYBR qPCR Master Mix (Vazyme) with gene-specific primers. The primers are listed in Table S2. The relative fold change in expression was calculated using the 2^−ΔΔC^_T_ method.

**CCK8 proliferation assay**

Equal numbers of mouse GCs were plated in a 96-well plate, and cell proliferation was measured with Cell Counting Kit-8 (CCK-8) (Vazyme, China). Briefly, CCK-8 reagent was added to each well of a 96-well plate and incubated with the GCs at 37°C for 2 h. The spectrophotometric absorbance at 450 nm of each sample was measured using a Synergy HTX Multi-Mode Reader (BioTek).

**Flow cytometry**

Annexin V FITC/PI staining was performed using an Annexin V-FITC Apoptosis Detection Kit (Beyotime, China) according to the manufacturers’ instructions. Briefly, GCs were washed with ice-cold PBS and then resuspended in 100 µL of binding buffer, followed by the addition of 5 µL Annexin V-FITC (20 mg/mL) and 10 µL PI (50 mg/mL). After incubation at room temperature for 15 min, 400 µL of binding buffer was added, and the cells were injected into a BD Accuri Cytometry (BD BioSciences, USA).

**Western blotting**

Total protein was extracted from the GCs using RIPA reagent (Solarbio, China), denatured, separated in a 10% polyacrylamide SDS‒PAGE gel, and transferred to a PVDF membrane (Millipore, USA). After blocking, the membrane was incubated with antibodies against BAX (1:2000, #2772, CST, USA), BCL2 (1:1000, ab196495, Abcam, USA), cleaved CASPASE3 (1:1000, AF7022, Affbiotech, China), cleaved CASPASE9 (1:2000, #9509, CST, USA), GAPDH (1:10000, ab181602, Abcam, USA), FOXQ1 (1:1000, sc-166265, Santa Cruz, USA), TSG101 (1:1000, A2216, ABclonal, China) and FLOT1 (1:1000, A6220, ABclonal, China) in Tris-buffered saline (TBS) containing 0.1% Tween-20 at 4 °C overnight. After 3 washes with Tris-buffered saline with 0.05% Tween-20, the membrane was incubated with a goat anti-rabbit or anti-mouse horseradish peroxidase immunoglobulin G (H+L) secondary antibody (1:5000, SA00001-2, Proteintech, China) at RT for 2 h. The protein bands were detected using a chemiluminescence detection kit (Servicebio, China) and a Bio-Rad Gel Imaging System (Bio-Rad, USA).

**Mitochondrial membrane potential measurement**

Mitochondrial membrane potential (MMP) depolarization was assessed using a mitochondrial membrane potential assay kit with JC-1 (Beyotime). Fluorescence images were captured with a Zeiss Axio Observer 5 fluorescence microscope (Carl Zeiss, Germany) and quantified according to the red to green fluorescence intensity ratio.

**ATP measurement**

The relative ATP content was measured using an ATP Assay Kit (Beyotime, China) according to the manufacturer’s instructions. Briefly, cells were lysed with ATP lysis buffer and centrifuged at 12,000 *g* and 4°C for 5 min. The working solution was added to a white opaque 96-well plate, after which the sample was added to the detection well, and the relative light unit (RLU) value was measured using a Synergy HTX Multi-Mode Reader (BioTek, USA).

**Relative mtDNA expression**

mtDNA was extracted from GCs using a TIANamp Genomic DNA Kit (TIANGEN, China). The relative expression of mtDNA was analyzed via qPCR using ND1 and *β*-globin primers. The primers used are listed in Table S2.

**Ovarian follicle counting**

The ovarian tissues of mice in the PBS and Y-exo injection groups were collected and fixed overnight in 10% buffered formalin for serial sectioning (5 μm) and hematoxylin and eosin and Masson staining. To evaluate the development of follicles in the two groups, follicles were counted every 5 consecutive sections.

**TUNEL assay**

Ovarian tissue was fixed with Bouin’s solution for 15 min, embedded in paraffin and sectioned consecutively. TUNEL assays were performed using a Roche™ TUNEL Assay Kit ™ according to the manufacturer’s instructions. Briefly, paraffin-embedded ovarian tissue sections were dewaxed and hydrated. The sections were treated with protease K working solution for 15–30 min at 37°C. Then, the sections were incubated with TUNEL Mixture at 37°C for 1 h. Images were observed with a microscope (Olympus, Japan).

**Immunofluorescence staining**

MII oocytes were collected from PBS- or Y-exo-treated female mice. Cumulus cells were removed using hyaluronidase for 3 min. Then, MII oocytes were fixed in 4% paraformaldehyde for 30 min, and 0.1% Triton-100 was added for 20 min. After blocking with 5% donkey serum for 1 h, MII oocytes were incubated with α-tubulin antibody (1:100, 66031-1-Ig, Affbiotech, China) at 4°C overnight and then with Alexa Fluor 488-labeled anti-mouse secondary antibody (1:250, SA00013-1, Affbiotech, China) for 1 h at room temperature. The nuclei were stained with DAPI for 10 min. MII oocytes were observed using confocal microscopy (Olympus, Japan).

**IVF and embryo culture**

Donor sperm was collected from Kunming male mice (13 weeks) into fertilization culture medium (K-SIFM-20, COOK) and incubated in oil for 1 h at 37°C with 5% CO_2_. Then, 10^5^/mL donor sperm were added to the fertilization culture medium together with MII oocytes. At 5 h after fertilization, the oocytes were picked and cultured in fertilization culture medium until the blastocyst stage. Two-cell embryos and blastocysts were recorded at 24 h and 96 h after fertilization.

**miRNA interference and overexpression**

Transfection of cells with siRNAs was performed using Lipofectamine 3000 (Thermo Fisher Scientific, USA) according to the manufacturer’s instructions. Cells were treated at 70% confluence with 100 nM miR-320-3p mimic or 200 nM miR-320-3p inhibitor or with their respective negative controls (RiboBio, China) for 24 h.

**Foxq1 interference and overexpression**

Cells were transfected with Foxq1-directed siRNA or the negative control (Sigma, USA) at 50 nM using Lipofectamine 3000 (Invitrogen, USA) for 24 h. The sequences used are provided as follows: si-Foxq1, 5'-GCCGGCGCGGCGCACCUGUdTdT-3'. For FOXQ1 overexpression, HEK293T cells were infected with lentivirus containing the packaging plasmids pMD2.G, psPAX2 or the lentiviral expression pCDH vector encoding GFP or GFP-FOXQ1 (Vigene Biosciences, China) for the production of lentiviral particles. Lentiviral particle-containing supernatants were collected from HEK293T cells at 24 and 48 h after infection, and titers were measured after concentration and purification. Lentiviral transduction for downstream experiments was accomplished at an MOI of 8.

**Luciferase reporter assays**

The FOXQ1 3'UTR containing miR-320a-3p target sequences was amplified with cDNA primers (Forward: 5'-GAAAG ATCGC CGTGT GACTC GAGGC AATTC TTGTT TTAGT TTCTT TGCGAA-3', Reverse: 5'-CCACT AATCC CTAGT TAGCC CGTTG CCTGC AACA-3') and cloned into the PGL3-CMV-LUC-MCS vector. The mutant FOXQ1 3'UTR was amplified with mutant cDNA primers (Forward: 5'-GAAAG ATCGC CGTGT GACTC GAGGC AATTC TTGTT TTAGT TTCTT TGCGA A-3', Reverse: 5'-CCACT AATCC CTAGT TAGCC CGTTG CCTGC AACA-3').

For the luciferase experiment, when the cell density was 70%, HEK293T cells were cotransfected with the luciferase reporter plasmid and miR-320a-3p control mimic/mimics using Lipofectamine 2000 (Invitrogen, USA). Fluorescence intensity was detected using a Dual Luciferase Reporter Gene Assay Kit (Genomeditech, China) after transfection for 48 h.

**Figure legends**

**Fig. S1. Characteristics of follicular fluid-derived exosomes.**

(A) Schematic diagram of the isolation and harvest of follicular fluid-derived exosomes. (B) A representative TEM image showing typical morphology of Y-exos and O-exos. (C–E) NTA was used to determine the size distribution and concentration of Y-exos and O-exos. (F) Follicular fluid-derived exosomes obtained from young (Y-exos) and old women (O-exos) were analyzed by Western blot with antibodies against exosome-specific markers TSG101 and FLOT1.

**Fig. S2. Y-exos can be internalized by GCs.**

(A) Identification of mouse ovarian GCs. The Red expressed FSHR, the blue expressed nuclear staining using 4',6-diamino-2-phenylindole (DAPI). Non-specific staining can be observed with PBS instead of primary or secondary antibodies. Scale bar, 100 μm. (B) TEM images of Y-exos. (C, D) NTA was used to determine the size distribution and concentration of Y-exos. (E) Western blot analysis showing the biomarkers of Y-exos, including TSG101 and FLOT1. (F) Representative images of Y-exos uptake by old mice derived GCs. Old mice derived GCs incubated with PKH67 labeled Y-exos for 24 h, and uptake of Y-exos was determined by fluorescence microscopy. Scale bar, 100 μm.

**Fig. S3. Effects of follicular fluid derived exosomes from young mice on the ovaries of aged mice.**

(A) Representative images of TUNEL assays on paraffin sections of control and Y-exos treated ovary. Scale bar, 50 μm. (B) Quantification of TUNEL positive cell counts in control and Y-exos treated ovary. *, *P* < 0.05, *n* = 5. (C) Relative expression of aging-related signaling molecules, and (D) telomerase-related genes in ovaries of each group. (E) Representative electron microscopy images of granulosa cells with or without Y-exos. (F) Abnormal mitochondrial number rates, (G) and lipid drops number from control and Y-exos treatment groups. *, *P* < 0.05. (H) Representative image and the ratio of red/green fluorescence intensity of granulosa cells mitochondrial membrane potential (Δ*Ψ*m) shown by JC-1 staining between control and Y-exos treatment groups. Red represents higher Δ*Ψ*m, green represents lower Δ*Ψ*m. Scale bar, 100 μm. (I) Relative ATP content in granulosa cells from control and Y-exos treatment groups. **, *P* < 0.01. (J) Relative expression of cell mtDNA in granulosa cells between two groups.

**Fig. S4. miR-320a-3p directly binds to FOXQ1 to regulate GCs proliferation.**

(A) *RPS6KB2*, *PPP2R5B*, *NR1H2* and *IGFBP5* mRNA quantitation by RT-qPCR in COV434 cells line after transfection with miR-320a-3p mimics, inhibitor or control. *, *P* < 0.05; **, *P* < 0.01; ***, *P* < 0.001; ****, *P* < 0.0001. (B) Schematic representation of putative miR-320a-3p binding sites on 3'UTR of PPP2R5B in human, mouse and rat. (C) COV434 cells line transfected with wild-type or mutated PPP2R5B 3'UTR luciferase constructs and control mimic or miR-320a-3p mimics. ****, *P* < 0.0001. (D) Schematic representation of putative miR-320a-3p binding sites on 3'UTR of IGFBP5 in human, chimp and rhesus. (E) COV434 cells line transfected with wild-type or mutated IGFBP5 3'UTR luciferase constructs and control mimic or miR-320a-3p mimics. ****, *P* < 0.0001.

**Fig. S5. Foxq1-siRNA promotes GCs proliferation.**

(A) RT-qPCR result of Foxq1 levels in cells transfected with control and siRNA. **, *P* < 0.01. (B) Western blot of FOXQ1 in cells transfected with control and siRNA. (C) Cell viability analyzed by CCK8 assay in control and Foxq1-siRNA. (D) Apoptotic rate of aged mice derived GCs was determined by flow cytometry. (E) Cleaved CASPASE-3, Cleaved CASPASE-9, BAX, and BCL2 protein expression were analyzed by Western blot. (F) Δ*Ψ*m assessed by JC-1 staining. Scale bar, 100 μm. (G) ATP concentration in aged mice derived GCs. *, *P* < 0.05. (H) Relative expression of cell mtDNA. *, *P* < 0.05.

**Table S1 Analysis of miR-320-3p-precipitated protein hnRNPA2B1**

|  | Granulosa cell cytoplasm (mi-320-3p-precipitated proteins) | Negative control (mi-320-3p-precipitated proteins) |
| --- | --- | --- |
| hnRNPA2B1 | 1 | 0 |
| hnRNPL | 1 | 0 |
| hnRNPR | 1 | 0 |
| hnRNPU | 1 | 0 |

hnRNPA2B1, heterogeneous nuclear ribonucleoprotein A2B1; hnRNPL, heterogeneous nuclear ribonucleoprotein L; hnRNPR, heterogeneous nuclear ribonucleoprotein R; hnRNPU, heterogeneous nuclear ribonucleoprotein U. Negative control: mouse cytoplasm extracts were incubated with non-labeled RNA control.

**Table S2 Primers sequence**

| Gene name | Primer sequences, 5’-3’ |
| --- | --- |
| Human *GAPDH* | F: GAAGGTGAAGGTCGGAGTC |
|  | R: GAAGATGGTGATGGGATTTC |
| Mouse *Actin* | F: GGCTGTATTCCCCTCCATCG |
|  | R: CCAGTTGGTAACAATGCCATGT |
| Mouse *p16* | F: GCTCAACTACGGTGCAGATTC |
|  | R: GCACGATGTCTTGATGTCCC |
| Mouse *Ccl20* | F: GCCTCTCGTACATACAGACGC |
|  | R: CCAGTTCTGCTTTGGATCAGC |
| Mouse *Mmp1* | F: CTTCTTCTTGTTGAGCTGGACTC |
|  | R: CTGTGGAGGTCACTGTAGACT |
| Mouse *Il-8* | F: CAAGGCTGGTCCATGCTCC |
|  | R: TGCTATCACTTCCTTTCTGTTGC |
| Mouse *P21* | F: CCTGGTGATGTCCGACCTG |
|  | R: CCATGAGCGCATCGCAATC |
| Mouse *Il-1α* | F: CGAAGACTACAGTTCTGCCATT |
|  | R: GACGTTTCAGAGGTTCTCAGAG |
| Mouse *P53* | F: GCGTAAACGCTTCGAGATGTT |
|  | R: TTTTTATGGCGGGAAGTAGACTG |
| Mouse *Mmp9* | F: CTGGACAGCCAGACACTAAAG |
|  | R: CTCGCGGCAAGTCTTCAGAG |
| Mouse *Tnks* | F: CCGTGTGTCTGTCGTTGAGTACC |
|  | R: CCATAGGAGCAGGCATTGTGAAGG |
| Mouse *Tert* | F: AAGACGAGCCTCACCTTCCAGAG |
|  | R: CTTCAACCGCAAGACCGACAGG |
| Mouse *Mre11a* | F: TCGGGCACAACATCTAGCAAACG |
|  | R: TCGGTTTCTTCTTGGGCAACTACTG |
| Mouse *Men1* | F: TGTGTGCCATCAACCCTTCCATC |
|  | R: TCCTCCAGGTCTGCCAAGTTCC |
| Mouse *Tep1* | F: CTGTGTAACCTGCTGCGGACTG |
|  | R: TCTGAATGGAAACTGCCGACTGTG |
| Mouse *Terf2* | F: CCTGGATGACACGGAGCCCTAC |
|  | R: GTTCTGAGGCTGTCTGCTTGGAG |
| Mouse *Foxq1* | F: GGCGGAGATCAACGAGTACC |
|  | R: GTACTCGCTGTTGGGGTTGA |
| Human *RPS6KB2* | F: GCGGCCTGTGGGACACTA |
|  | R: GTTGGTGCCTTGCACCTTTC |
| Human *PPP2R5B* | F: CCCAGTGTGGGGTGATGTTT |
|  | R: TCATGCGGATGATGTCTGGG |
| Human *NR1 H2* | F: CAGAGCGCAAGCGAAAGAAG |
|  | R: GCTGAGCACGTTGTAGTGGA |
| Human *FOXQ1* | F: GGCGGAGATCAACGAGTACC |
|  | R: GTACTCGCTGTTGGGGTTGA |
| Human *IGFBP5* | F: AACGAAAAGAGCTACCGCGA |
|  | R: CCGACAAACTTGGACTGGGT |
| Mouse *Nd1* | F: CCCTAAAACCCGCCACATCT |
|  | R: GAGCGATGGTGAGAGCTAAGGT |
| Mouse *β-globin* | F: AAAGGTGCCCTTGAGGTTGTC |
|  | R: TGAAGGCTCATGGCAAGAAA |

1. Liu Y, Shen Q, Li H, et al. Cell-free mitochondrial DNA increases granulosa cell apoptosis and reduces aged oocyte blastocyst development in the mouse. *Reprod Toxicol* 2020;98:278–85.
